# Outcome Probability versus Magnitude: When Waiting Benefits One at the Cost of the Other

**DOI:** 10.1371/journal.pone.0098996

**Published:** 2014-06-03

**Authors:** Michael E. Young, Tara L. Webb, Jillian M. Rung, Anthony W. McCoy

**Affiliations:** 1 Kansas State University, Manhattan, Kansas, United States of America; 2 Southern Illinois University, Carbondale, Illinois, United States of America; 3 Utah State University, Logan, Utah, United States of America; University of Chicago, United States of America

## Abstract

Using a continuous impulsivity and risk platform (CIRP) that was constructed using a video game engine, choice was assessed under conditions in which waiting produced a continuously increasing probability of an outcome with a continuously decreasing magnitude (Experiment 1) or a continuously increasing magnitude of an outcome with a continuously decreasing probability (Experiment 2). Performance in both experiments reflected a greater desire for a higher probability even though the corresponding wait times produced substantive decreases in overall performance. These tendencies are considered to principally reflect hyperbolic discounting of probability, power discounting of magnitude, and the mathematical consequences of different response rates. Behavior in the CIRP is compared and contrasted with that in the Balloon Analogue Risk Task (BART).

## Introduction

Life invariably involves trade-offs. Gaining one product feature may come at the loss of another if the cost of the product is to remain constant, or both features can be obtained if an increase in cost is tolerable. A dollar in the pocket is a sure gain but must be considered against the probability of obtaining much more if spent on a lottery ticket. A new HDTV can be purchased with the money in one's savings account, or this money could be invested at a 5% interest rate to create greater value but at a significant delay. Understanding how people make these trade-offs involves the assessment of the relative importance of factors like absolute magnitude, probability, and temporal proximity and the calculus driving their integration [1].

Prior research in probability discounting has examined the trade-off between magnitude and probability by studying the preference for smaller certain outcomes versus larger uncertain outcomes [2]–[4]. Research on delay discounting (aka temporal discounting) has examined the trade-off between magnitude and temporal proximity by studying the preference for smaller sooner outcomes versus larger later outcomes [3], [5]–[8]. The present project is part of a series of studies in which we are examining behavior when magnitude and probability are changing over time thus requiring the integration of preferences for higher magnitude, higher probability, and shorter delays.

A conventional approach to addressing the question of relative importance would present participants with a series of static choices, much like those used in most studies of probability and delay discounting [1]. “Would you rather have a 35% chance of winning $100 in a week or a 10% chance of winning $2,000 in a month?” After a series of such choices, a multidimensional map of the decision space could be created in order to describe the relative trade-off among these decision factors. Our interest, however, is in how these decisions are managed within the context of events unfolding in time where the dynamics of value are in flux. The nature of a reward may change over time – the longer you wait, the less pie is available or the likelihood of the store having the item in stock may decrease. Thus, many choice situations are dynamically changing and require a series of choices between taking what is available now or waiting for a higher likelihood or magnitude. These choices are even more challenging when waiting might increase one attribute (e.g., magnitude of the reward) while decreasing another (e.g., the probability of obtaining the reward).

In a prior project, Young, Webb, and Jacobs [9] assigned participants to either a condition in which probability increased over time or magnitude increased over time. Participants were playing a customized first-person-shooter video game in which waiting for up to 10 s between shots could increase the damage potential of their weapon, either in the form of a higher probability of firing or a greater magnitude of damage. They reported that participants waited longer to obtain a higher probability of an outcome than they did when waiting for the equivalent magnitude. For example, players in the video game were much more likely to wait to obtain a 70% chance of doing 100 points of damage than they were to wait for 70 points of damage, an outcome that matches the first in expected value. This result suggests that having a high probability of an outcome is more important than an equivalent magnitude and thus is reminiscent of the certainty effect [4].

In a recent study, Webb and Young (submitted) manipulated probability and magnitude within-participant so that both were increasing across time but at identical (Experiment 1) or different (Experiment 2) rates. Webb and Young found much greater waiting to obtain a higher probability than to obtain a higher magnitude when each was manipulated in isolation, and behavior when both increased together was more similar to the condition in which only probability changed (Experiment 1). They also reported greater sensitivity to changes in probability than to changes in magnitude (Experiment 2). Thus, reward probability was having a stronger effect on decisions to wait than an equivalent reward magnitude.

The most commonly used task to study the trade-off between probability and magnitude across time is the Balloon Analogue Risk Task ([or BART, [10]). The BART involves changes in probability and magnitude across time, but in this task the two factors are arrayed in opposition – the likelihood of earning a reward decreases with time as the magnitude of the outcome increases. Specifically, pumping up a virtual balloon increases its size (which corresponds to the magnitude of the reward available), but this increase in magnitude comes at the risk of obtaining no reward if the balloon bursts on the next pump. Thus, participants must trade off the increase in magnitude against the decrease in probability. In the BART, participants routinely stop pumping much earlier than is optimal [10], [11]. As in the Young et al. (2011) and Webb and Young (submitted) studies, this behavioral tendency may reflect greater weight given to probability than to magnitude – participants cash in sooner than they should because they prefer the higher probability of a lesser amount to the lower probability of a greater amount.

Unlike the Young et al. [9] video game task, the issue of delay is only tangentially a factor in the BART. In the Young et al. video game, faster responding produces more opportunities to respond and task completion depends on accumulating a minimum amount of the damage on the targets within the time allotted. For the BART, participants receive a predetermined number of choices such that cashing in sooner does not produce an increase in the number of opportunities to choose. However, from the perspective of the participant, the opportunity to terminate the experiment sooner rather than later may create an incentive to trade off time against magnitude and probability [12]. If cashing in too early (relative to optimal) results in a 50¢ reduction in money earned by the end of the study but allows one to leave 20 minutes early, the trade-off may be worthwhile to the person performing the task. A tendency to cash in early may also be a product of the pumping of the balloon which requires a large number of responses (usually mouse clicks) that can produce a degree of response fatigue. Furthermore, the BART and the CIRP also differ in other ways including the explicit reward (money vs. damage to target), the surface task characteristics (inflating a balloon vs. destroying targets), the way in which magnitude and probability are communicated (balloon size for magnitude and number of pumps for probability vs. charge bar level for both magnitude and probability), inter alia. Given their functional similarity, behavior in the BART suggests certain outcomes may be observed in the CIRP and vice versa – if these other task differences are secondary to those driving behavior.

If the generally conservative behavior in the BART indeed reflects an aversion to risk rather than a trade-off of money for time or fatigue, what if waiting longer increased the probability of obtaining an outcome but at a cost in magnitude? Under these conditions, a participant might be expected to wait longer than is optimal in order to obtain a higher probability of a smaller magnitude. This behavior may indeed cost the participant time without an associated increase in actual payout. Unfortunately, the cover story of the BART is rooted in the everyday experience of blowing up a balloon. Having a balloon that shrinks in size with an increasingly lower probability of popping does not map well to the ecological framing of these experiences. Furthermore, the BART cannot be redesigned to start with a very high probability of popping because doing so would truncate most trials before a player could progress (i.e., if most balloons pop quickly, there is no opportunity to decrease its size in order to obtain a higher probability of obtaining the reward). Thus, we adapted Young et al.'s [9] and Webb and Young's (submitted) video game platform (which we will call the Continuous Impulsivity and Risk Platform or CIRP) to investigate this trade-off. The CIRP has a secondary benefit in that magnitude and probability automatically change over time without the need to pump, thus reducing the effect of fatigue on decisions to cash in.

In Experiment 1, a small change in the cover story created a plausible scenario in which waiting increased the likelihood of a weapon working but at a cost in the amount of damage that could be produced. If participants were cashing in too early in the BART (relative to optimal) in order to avoid risk, then players in our game should cash in too late because they are seeking a higher probability at the cost of a lower magnitude.

In Experiment 2, we used the CIRP to re-examine the contingencies present in the BART where magnitude increased across time while probability decreased. This design served to confirm that participants would cash in too early in the CIRP as they did in the BART. Importantly, the design of Experiment 2 also discriminated between two alternative hypotheses of the behavior observed in Experiment 1 regarding whether or not the participants were sensitive to the opportunity to make more choices by waiting less.

## Experiment 1

In order to investigate the trade-off between magnitude and probability, magnitude began at maximum and steadily decreased across a 10 s interval until reaching a value of zero. In the game, the available magnitude is indicated by a charge bar to the left of the middle of the screen. At the same time that magnitude was decreasing, probability steadily increased during the same period, beginning at a 0% and increasing to 100% at the end of the 10 s interval. A second charge bar adjacent to the first indicated the probability. The changes in the charge levels mirrored one another; for example, a 1 cm drop in the magnitude charge bar was accompanied by a 1 cm increase of the probability charge bar.

**Figure 1:**
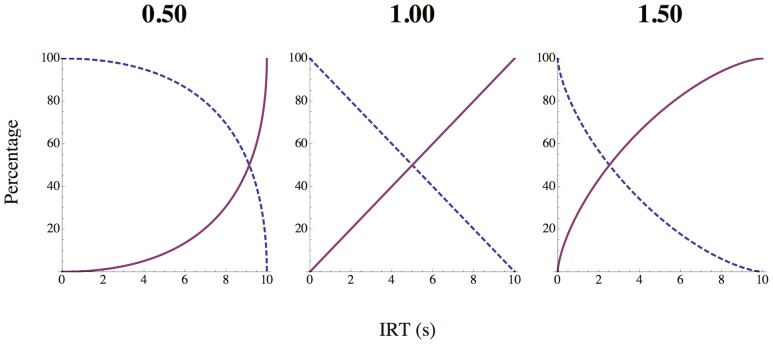
Changes in magnitude and probability across time. The effects of three values of the power parameter of Equation 1 on the rate of change in magnitude and probability over the recharge interval. For Experiment 1, the dashed lines represent the magnitude of damage and the solid lines represent the probability of a successful shot. The opposite is true for Experiment 2.

The mathematical relationship that defined the rate at which the probability increased was:
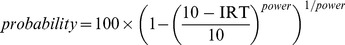
(1)where *IRT* is the time waited between shots (the interresponse time), and *power* is a parameter that defines the rate of change; the probability could not increase beyond 100%. Because magnitude was programmed to mirror probability, the proportion of available magnitude at time *t* was one minus the current probability/100. Figure 1 illustrates three values of the power parameter and their effect on the rate of change in magnitude and probability. For low values of power, both follow a positively accelerating function but in opposite directions. For high values of power, both follow a negatively accelerating function. For a power of 1.00, the relationship is linear.

**Figure 2:**
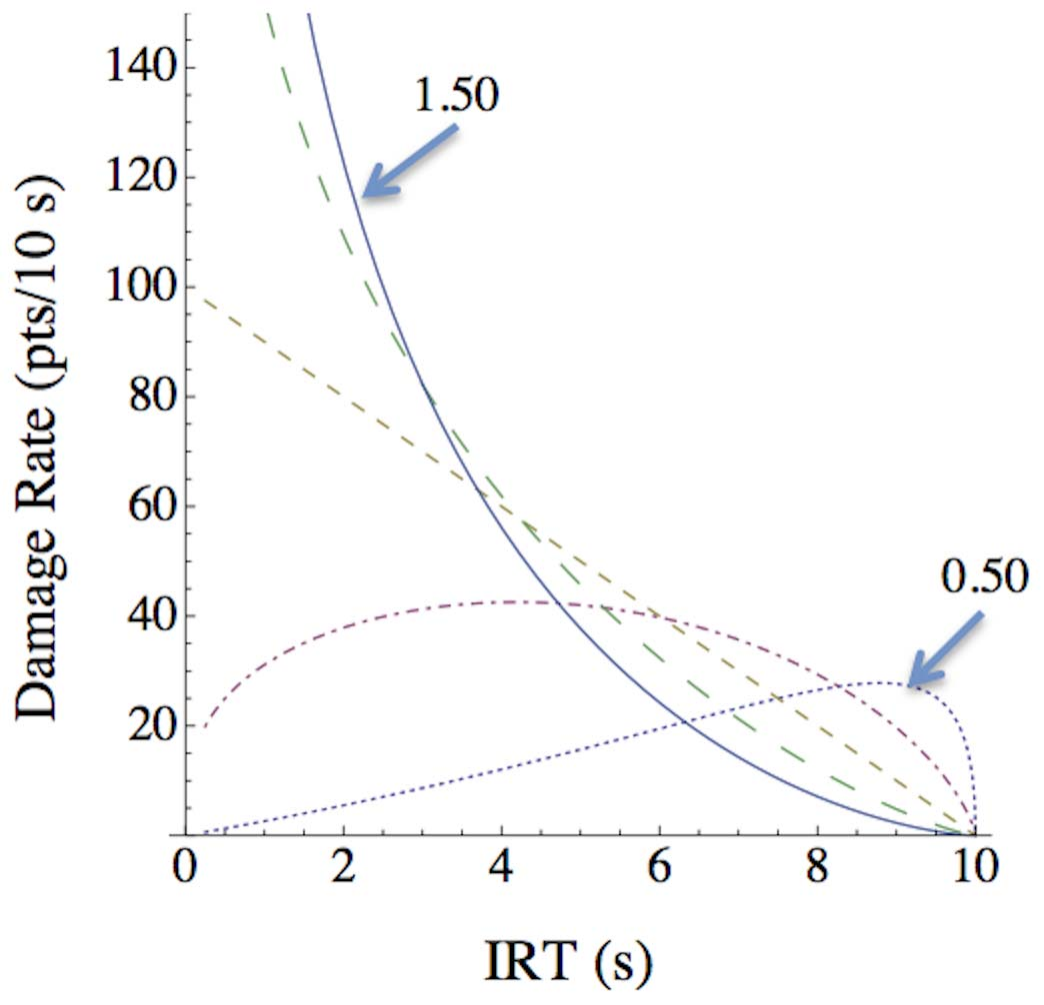
Damage produced for specified power values as a function of time waited. Rate of damage per 10

Figure 2 illustrates the rate of damage that would be produced for a consistent IRT at five representative power values. The calculation of damage rate for a consistent IRT incorporates both the expected value of damage done for a single shot as well as the advantages of firing faster:
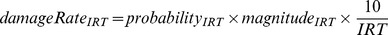
(2)where *probability* is the probability of obtaining the outcome for a given IRT (as specified in Equation 1), *magnitude* is the magnitude of outcome for that IRT (the inverse of Equation 1), and the 10/IRT term incorporates the relative advantage of firing faster versus slower. For example, if the probability at an IRT of 2 s is .90 and the magnitude is 10 points, the damage rate is .90×10×10/2 = 45 points per 10 s. If the probability at an IRT of 5 s is .40 and the magnitude is 60, the damage rate is .40×60×10/5 = 48 points per 10 s.

When Equation 2 is applied to the types of designs shown in Figure 1, for low power values the participants would destroy the targets at a faster rate if they waited between shots. For high power values, the participants would destroy the targets at a faster rate if they responded as quickly as possible (approximately 0.25 s between shots). Thus, the optimal IRT shifts as the power value changes. If participants are placing more emphasis on a higher probability than a higher magnitude (consistent with Young et al., 2011), then they should wait longer than is optimal in order to obtain the higher probabilities available toward the end of the 10 s interval.

Consistent with prior work using the video game to study impulsivity, we expected that participants would vary both in their overall wait time and in their sensitivity to the changes in the power value. Thus, we revisited the ability to predict individual behavior by considering sex, prior video game play, and a common measure of trait impulsivity [13] as predictors of both the trade-off between probability and magnitude as well as their sensitivity to the contingencies present in the task. Previous studies have found small and inconsistent individual differences in experiments using the CIRP [9], [14], [15]. Similarly, research using the BART has inconsistently found differences based on sex and impulsivity [16]–[19]. When found, the individual differences have been broadly consistent with the conceptual understanding of the impulsivity construct, but these studies and the present experiments were not designed to maximally assess individual differences (e.g., by using large sample sizes or systematic sampling across the full range of the predictors to avoid restriction of range). Regardless, we will continue to examine the predictive efficacy of sex, prior video game experience, and measures of impulsivity in order to lay the groundwork for a focused study of individual differences in CIRP tasks.

### Method

#### Participants

A total of 76 students enrolled in an introductory psychology course at Southern Illinois University served as participants in Experiment 1 and received course credit for their voluntary participation. The study was approved by the Southern Illinois University Institutional Review Board and written informed consent was obtained from all participants. There were 34 males and 42 females.

#### Procedure

The Torque Game Engine (obtained from www.garagegames.com) was used to develop the video game. The video game environment was programmed to include four visually identical levels consisting of seven separate regions, each populated with two stationary orcs. Each orc faced a village building and fired shots at the building every 4 s on average. In each 2-orc region, the firing of one of the orcs produced contingent explosions; this design was used to make the player's goal more realistic. The player's task was to effectively destroy all of the orcs within each game level. Slightly to the left of the center of the participant's screen, there was an orange bar that corresponded to the damage magnitude and a green bar that corresponded to the probability of successfully firing a shot; these bars showed the participant the current value of each outcome attribute. The orange bar was labeled “0 pts” on the bottom and “100 pts” on the top, and the green bar was labeled “0%” on the bottom and “100%” on the top. After each shot, the orange bar was refilled and gradually emptied over a 10 s period whereas the green bar emptied and gradually filled over a 10 s period. A movie clip with the probability decreasing while magnitude increased (as tested in Experiment 2) is available at: http://www.k-state.edu/psych/research/young/suppmaterial.html.

The power parameter's value (and hence the behavior of the player's weapon charge) changed each time the participant destroyed two orcs. Therefore, each player experienced seven power values per level. We used a random sampling design [20] to choose each new power value in the 0.50 to 1.50 range (uniformly sampled). This range of values was used to ensure that participants experienced values at which it was optimal to wait (those less than 1.00) and values at which it was optimal to fire as rapidly as possible (those greater than 1.00) (see Figure 2). The main advantage of using the random sampling design was that it allows the results to generalize to the entire range of the functional relationship and to identify the nature of the relationship. A three-tone sequence made up of three 250 ms pure tones each separated by 250 ms of silence (for a total duration of 1250 ms) played each time the weapon power changed thus signaling the change.

If a shot was unsuccessful, the crossbow would fire a projectile that failed to explode, therefore dealing no damage to the target. It took 2.7 successfully fired full-charge shots to eliminate a target. Given that a full-charge shot would have a 0% chance of success (and only a 0 damage shot has a 100% chance of success), the median number of shots observed to destroy a target was much larger – 10.

After completing the video game task, students were asked to complete the UPPS-P impulsive behavior scale [21] and demographic questions indicating their sex and detailing their previous video game experience to determine if there were any individual differences in impulsivity in the video game task that could be accounted for by responses on these measures. All of the aforementioned questionnaires were completed via a program created in PsyScope [22].

The dependent measure was interresponse time (IRT). IRTs greater than 20 s were dropped from the analysis, because they were likely due to prolonged travel time or inattention. In previous CIRP studies, approximately 2% of responses were omitted using this criterion [9]. The IRT variable was dichotomized for the purposes of the analysis, because IRTs were bimodal. Therefore, IRTs greater than 5 s and less than 20 s were coded as a “1” to designate waiting, whereas IRTs less than 5 s were coded as a “0” to designate not waiting. This choice was motivated by the value being in the middle of the typical 0 to 10 s range of IRTs and to be consistent with our other published results using this procedure.

### Results

Of the 76 participants, 3 did not proceed past the first level, but all participants were retained for the analysis. The exclusion of IRTs greater than 20 s resulted in excluding fewer than 3% of the responses.

#### Waiting Time

The data were analyzed with a linear mixed effects (i.e., multilevel or random coefficients) analysis using *R*'s lmer function and specifying a binomial error distribution [23]. This approach can be conceptualized as a repeated measures logistic regression. A mixed effects analysis uses maximum likelihood estimation to address missing data and the highly unbalanced data from this free operant task, plus the analysis allows the fitting of individual regression curves for participants who (by design) did not receive the same set of power values. The power variable was centered to avoid multicollinearity issues because both main effects and the interaction were examined in the model. In order to determine the best fitting model for predicting the likelihood of waiting, the fixed effects of power, game level, and their interaction were evaluated. Random effects of intercept, power, and game level were examined to allow individual variation in overall waiting, sensitivity to power, and learning, respectively.

**Figure 3:**
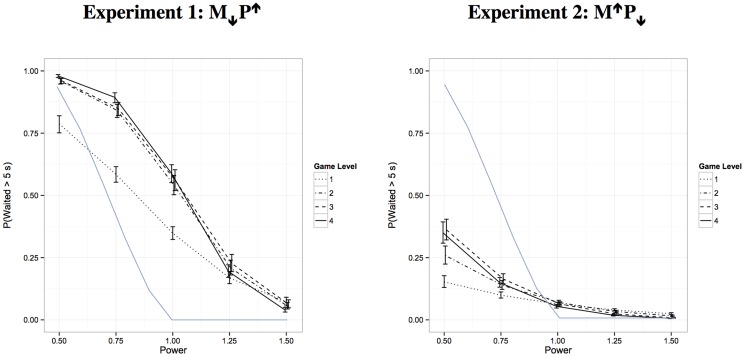
Results of Experiments 1 and 2. Probability of waiting at least 5-fitting multilevel regression model as a function of power (see Equation 1) and game level or block of experience. The solid line without error bars represents optimal waiting under the assumption that an optimal wait time of m seconds would produce a m/10 probability of waiting more than 5 seconds.

The complete model provided the best fit as determined by the Bayesian Information Criterion or BIC. The model fits are shown in Figure 3 along with a superimposed optimal waiting time curve with the assumption that the optimal time (e.g., 7 s) would roughly translate into an equivalent percentage likelihood of waiting more than 5 s (e.g., 70%). Participants waited much longer than is optimal, and the degree of suboptimality increased after the first level. Sensitivity to the changing incentives for waiting (i.e., changes in the power value) is evidenced by the slope of each line. Participants showed very strong sensitivity, and this sensitivity appeared to increase across game levels. Note that a game level is functionally equivalent to a block of training and thus variation across levels represent behavioral change as a result of learning.

The mixed effects analysis confirmed strong sensitivity to power in the first level (slope = −3.87, *z* = −9.35 for comparison to zero slope, *p*<.01) that increased in the second and third levels (slopes  = −6.00 and −5.90, *z*s = −10.31 and −9.72, *p*s<.01, for comparisons to first level), and increased again by the final level (slope  = −7.10, *z*s>−2.66, *p*s<.01, for comparisons to second and third levels). The overall likelihood of waiting at the average power value (1.00) increased between the first level (35%) and subsequent levels (54%, 56%, and 59%, *z*s>5.05, *p*s<.01, for all three comparisons) but did not differ across the final three levels (*z*s<0.23).

#### Predicting Individual Differences

After identifying the best model, we considered the individual differences variables of sex, video game play, and UPPS-P scores. Because the relatively small number of participants in each condition does not support a thorough individual differences analysis, these results should be considered exploratory. Each variable was considered as a main effect and in an interaction with the power variable without the presence of the other variables in order to avoid multicollinearity. We did not correct for multiple comparisons, thus this exploratory approach is prone to Type I errors. The Bayesian Information Criterion (BIC) was used to determine if a model including one of the individual differences variables improved the best fit.

The UPPS-P lack of premeditation score was the only demographic variable that reached statistical significance. A lack of premeditation has been defined as the inability to think through possible consequences of one's behavior before acting. Participants with higher scores on lack of premeditation tended to have longer IRTs, but not significantly so (*z* = 1.43, *p* = .15), and somewhat greater sensitivity to power (*z* = −2.78, *p*<.01). In the latter case, the greater sensitivity reflects behavior that looks more like performance in the latter levels of the game (see Figure 3) and thus does not reflect greater optimality.

### Discussion

When the contingencies were arrayed such that probability increased while magnitude decreased, participants waited much longer than is optimal. This tendency is opposite that observed in the BART where probability decreases while magnitude increases. Although the two tasks differ in many other ways, this key difference suggests that the early responding in the BART and the later responding in the CIRP may reflect a desire to obtain a higher probability, even if doing so results in sacrificing magnitude in a suboptimal way or when doing so lengthens the task as it did in Experiment 1.

Only one of our individual differences predictors reached statistical significance. Interestingly, Miller, Flory, Lynum, and Leukefeld [24] documented that lack of premeditation on the UPPS most commonly produced the strongest correlation with externalizing problems like conduct problems, alcohol and drug use, ADHD, and risky sex. In Experiment 1, behavior was related to lack of premeditation in the form of greater sensitivity to the power manipulation which, when coupled with the slightly longer IRTs evidenced by those with a greater lack of premeditation, was not indicative of greater optimality. Given that this is the first indication of an association with a UPPS score and behavior in the game, the result should be treated with caution.

Because the BART and our opposition task implemented in the CIRP differ along a number of dimensions, in Experiment 2 we decided to create a conceptual replication of the BART contingencies by having probability decrease while magnitude increases in the CIRP. If participants are indeed responding earlier in the BART and later in Experiment 1 due to a desire to obtain higher probability while sacrificing magnitude, then we should observe much shorter wait times than is optimal in Experiment 2. If another strategy is used that is insensitive to the differences in probability and magnitude (e.g., perhaps people in Experiment 1 are simply waiting until the bars are roughly the same height), then observed behavior in Experiments 1 and 2 would be indistinguishable.

## Experiment 2

The second experiment replicated the contingencies present in the BART where magnitude increased with time while probability simultaneously decreased. This served to explore alternative hypotheses of behavior and sensitivity to the manipulation of the power parameter.

### Method

#### Participants

A total of 55 students enrolled in an introductory psychology course at Southern Illinois University served as participants in Experiment 2 and received course credit for their voluntary participation. The study was approved by the Southern Illinois University Institutional Review Board and written informed consent was obtained from all participants. There were 38 males and 17 females.

#### Procedure

The procedure in Experiment 2 was identical to that of Experiment 1, with the exception that the nature of the two charge bars reversed: the orange charge bar depicting magnitude gradually filled over 10 s and emptied after every shot, and the green bar depicting the probability of the weapon firing gradually emptied over 10 s and refilled after every shot. All participants completed the same series of questionnaires as described in Experiment 1 after completion of the video game task.

### Results

Of the 55 participants, all participants proceeded to at least the third level of the game and were retained for the analysis. The exclusion of IRTs greater than 20 s resulted in excluding fewer than 1% of the responses.

#### Waiting Time

The same analytical approach used in Experiment 1 was used here. The complete model again provided the best fit as determined by the BIC. The model fits are shown in Figure 3 along with a superimposed optimal waiting time. Participants waited much less than is optimal, but the degree of suboptimality decreased after the first level. Participants showed good sensitivity to the different incentives for waiting created by changing the power value, and this sensitivity appeared to increase across game levels.

The mixed effects analysis confirmed good sensitivity to power in the first level (slope  = −1.95, *z* = −7.61, *p*<.01, for comparison to zero slope) that increased in the second level (slope  = −3.07, *z* = −5.17, *p*<.01, for comparison to first level), and increased again in the final two levels (slopes  = −4.22 and −4.47, *z*s = −4.39 and −5.33, *p*s<.01, for comparisons to second level). The overall likelihood of waiting at the middle power value (1.00) did not significantly differ across the four levels (6%, 7%, 6%, and 5%, *z*s<1.01, for all comparisons).

#### Predicting Individual Differences

After identifying the best model, we considered the individual differences variables of sex, video game play, and UPPS-P score in the same manner as we did for Experiment 1. None of the individual differences variables predicted the overall tendency to wait nor the sensitivity to the power manipulation.

## General Discussion

When facing a trade-off between obtaining a higher probability of an outcome versus a higher absolute magnitude, each of which is changing over time, participants showed a strong tendency to prefer higher probabilities over higher magnitudes. When probability was initially low and magnitude was high, waiting longer increased the probability of the outcome but decreased the magnitude obtainable (Experiment 1). Under these conditions, participants waited much longer than optimal even though doing so lengthened the experiment. When probability was initially high and magnitude was low, waiting longer decreased the probability of the outcome but increased the magnitude obtainable (Experiment 2). Under these conditions, participants waited much less than optimal, again lengthening the experiment. Given the generally consistent behavior across our Experiment 2 and the published BART results, the results confirm that secondary task characteristics (the nature of the reward, the balloon vs. game task, etc.) may not be critical to the waiting behavior observed.

Why might participants be predisposed toward whatever behavior produced a higher probability of an outcome while sacrificing its obtainable magnitude? Webb and Young (submitted) proposed that the hyperbolic discounting of probability in an experience-based decision making paradigm [3] would emphasize small differences in probability at the upper end of the probability scale. This emphasis might produce greater perceived benefits of a behavior that keeps the probability near 1.00. Conversely, Webb and Young noted that the impact of increases in magnitude often fall off logarithmically or approximately so [25], [26]. Thus, behaviors that produce changes at the lower end of the magnitude scale are predicted to have a bigger impact than changes at the upper end of the scale. When the hyperbolic discounting of probability is combined with a logarithmic-like discounting of magnitude, behavior will be produced that reflects an oversensitivity to changes in probability near 1.00, and an undersensitivity to changes in higher magnitudes.

Unlike behavior using the CIRP, there appears to be no benefit to cashing in quickly in the BART because time-on-task is not based on the amount of reinforcement earned (money in the BART, weapon damage in the CIRP) but on the number of times the person cashes in (number of balloons in the BART). The BART, however, may still contain an incentive to respond more impulsively by taking the smaller sooner reward in order to shorten the task duration and/or to avoid fatigue due to the need to pump up the balloon. Regardless of these task differences, both procedures appear to produce qualitatively similar tendencies to seek out greater probability of reinforcement at the cost of optimal total reinforcement. It is not clear, however, whether participants performing the BART might wait longer (although still cash in too early) if the task were modified to either (a) change the goal to achieving a target amount of money by dynamically changing the number of possible balloons or (b) reduce fatigue by eliminating the pumping requirement ([27], however, developed an automatic BART for which participants still responded too early, even when told the optimal number of pumps]). It is interesting to note that the blue balloons in Lejuez et al. [10] that required the most pumps to achieve optimal performance, 64, produced proportionally less waiting before cashing in (about 28 pumps or 44% of optimal) than the yellow and orange balloons that required considerably fewer responses for optimal performance (16 and 4, respectively). The yellow and orange balloons also produced proportionally longer waits before cashing in (about 11 and 3.5 responses or about 69% and 82% of optimal, respectively).

### Final Thoughts

Although participants examined using the CIRP differ significantly in both their overall tendency to wait and their sensitivity to the differential consequences for doing so [9], [14], [15], these studies have shown that individual differences on behavioral tasks tend to be (at best) weakly related to self-report measures of impulsivity like the UPPS-P and the Barratt Impulsiveness Scale [13]. These weak relationships may reflect a lack of statistical power, restricted range, or the limited behavioral observation period in behavioral assessments like the CIRP, BART [10], Experiential Discounting Task [8], and Immediate Memory Task and other related measures [28]. However, the results may also indicate that these behavioral assessments are more sensitive to state variables (e.g., attentiveness, recent consumption of caffeine or glucose, distractions during testing, anxiety) thus overshadowing the effects of trait variables like those assessed by self-report measures [8]. Given that correlations between self-report and behavioral measures of impulsivity are usually below .35, it would behoove the field to consider the within-subject assessment of behavior across a much wider range of conditions to determine the extent to which these correlations might increase when behavior is collapsed across variations in state.

Even in the presence of strong disincentives, participants responded either more quickly or slowly than is optimal in order to obtain a high probability of the outcome while sacrificing expected value over time. In questionnaire assessments, there is no cost to suboptimal choice because the outcomes are not experienced. In the BART, there is a hidden incentive to respond more rapidly because it allows the participant to finish the task more quickly and to reduce response fatigue. Although doing so may sacrifice monetary payoff in some experiments, the time saved may be more valuable if the payment differential is relatively small (as it usually is in the BART). In our opposition study using the CIRP, not only did suboptimal behavior lengthen the task, but the degree of suboptimality did not change substantially over the hour-long experiment that involved hundreds of responses and their consequences. Clearly, a desire for certainty in the context of our experiments is detrimental and resistant to change, at least in the presence of gains [4]. It is possible that the contingencies present in laboratory-based behavioral assessments may not reflect the contingencies that exist in everyday choice and thus behavior may reflect extra-experimental experience where a desire for certainty is normative. However, the modern world is replete with environments where an undue aversion to immediate risk produces poorer long-term outcomes (e.g., in personal investments, product development, vaccinations, or sports). Thus, this desire for certainty in the face of gain instead may reflect something much deeper in the human psyche produced by more basic evolutionary pressures [29], [30]. If so, an hour-long experiment is clearly insufficient to shift this instinctual desire.

## References

[1] Weber BJ, Chapman GB (2005) The combined effects of risk and time on choice: Does uncertainty eliminate the immediacy effect? Does delay eliminate the certainty effect? Organizational Behavior & Human Decision Processes 96: 104–118.

[2] Shead NW, Hodgins DC (2009) Probability discounting of gains and losses: Implications for risk attitudes and impulsivity. Journal of the Experimental Analysis of Behavior 92: 1–16. 20119519 10.1901/jeab.2009.92-1 PMC2707142

[3] Rachlin HC, Raineri A, Cross D (1991) Subjective probability and delay. Journal of the Experimental Analysis of Behavior 55: 233–244. 2037827 10.1901/jeab.1991.55-233 PMC1323057

[4] Kahneman D, Tversky A (1979) Prospect theory: An analysis of decisions under risk. Econometrica 47: 313–327.

[5] Green L, Myerson J (2004) A discounting framework for choice with delayed and probabilistic rewards. Psychological Bulletin 130: 769–792. 15367080 10.1037/0033-2909.130.5.769 PMC1382186

[6] Chapman GB (1995) Valuing the future: Temporal discounting of health and money. Medical Decision Making 15: 373–388. 8544681 10.1177/0272989X9501500408

[7] Loewenstein G (1988) Frames of mind in intertemporal choice. Management Science 34: 200–214.

[8] Reynolds B, Richards JB, de Wit H (2006) Acute-alcohol effects on the Experiential Discounting Task (EDT) and a question-based measure of delay discounting. Pharmacology, Biochemistry and Behavior 83: 194–202. 10.1016/j.pbb.2006.01.007 16516954

[9] Young ME, Webb TL, Jacobs EA (2011) Deciding when to “cash in” when outcomes are continuously improving: An escalating interest task. Behavioural Processes 88: 101–110. 21871951 10.1016/j.beproc.2011.08.003 PMC3523357

[10] Lejuez CW, Read JP, Kahler CW, Richards JB, Ramsey SE, et al (2002) Evaluation of a behavioral measure of risk taking: The Balloon Analogue Risk Task (BART). Journal of Experimental Psychology: Applied 8: 75–84. 12075692 10.1037//1076-898x.8.2.75

[11] Wallsten TS, Pleskac TJ, Lejuez CW (2005) Modeling behavior in a clinically diagnostic sequential risk-taking task. Psychological Review 112: 862–880. 16262471 10.1037/0033-295X.112.4.862

[12] Paglieri F (2013) The costs of delay: Waiting versus postponing in intertemporal choice. Journal of the Experimental Analysis of Behavior. 10.1002/jeab.18 23413105

[13] Patton JH, Stanford MS, Barratt ES (1995) Factor structure of the Barratt impulsiveness scale. Journal of Clinical Psychology 51: 768–774. 8778124 10.1002/1097-4679(199511)51:6<768::aid-jclp2270510607>3.0.co;2-1

[14] Young ME, Webb TL, Sutherland SC, Jacobs EA (2013) Magnitude effects for experienced rewards at short delays in the escalating interest task. Psychonomic Bulletin and Review 20: 302–309. 23188742 10.3758/s13423-012-0350-7 PMC3594385

[15] Young ME, Webb TL, Rung JM, Jacobs EA (2013) Sensitivity to changing contingencies in an impulsivity task. Journal of the Experimental Analysis of Behavior 99: 335–345. 23658118 10.1002/jeab.24 PMC3900408

[16] Aklin WM, Lejuez CW, Zvolensky MJ, Kahler CW, Gwadz M (2005) Evaluation of behavioral measures of risk taking propensity with inner city adolescents. Behavior Research and Therapy 43: 215–228. 10.1016/j.brat.2003.12.007 15629751

[17] Hopko DR, Lejuez CW, Daughters SB, Aklin AW, Osborne A, et al (2006) Construct validity of the Balloon Analogue Risk Task (BART): Relationship with MDMA use by inner-city drug users in residential treatment. Journal of Psychopathology and Behavioral Assessment 28: 95–101.

[18] Hunt MK, Hopko DR, Bare R, Lejuez CW, Robinson EV (2005) Construct validity of the Balloon Analog Risk Task (BART): Associations with psychopathy and impulsivity. Assessment 12: 416–428. 16244122 10.1177/1073191105278740

[19] Lejuez CW, Aklin W, Daughters S, Zvolensky M, Kahler C, et al (2007) Reliability and validity of the youth version of the Balloon Analogue Risk Task (BART-Y) in the assessment of risk-taking behavior among inner-city adolescents. Journal of Clinical Child and Adolescent Psychology 36: 106–111. 17206886 10.1080/15374410709336573

[20] Young ME, Cole JJ, Sutherland SC (2012) Rich stimulus sampling for between-subjects designs improves model selection. Behavior Research Methods 44: 176–188. 21761262 10.3758/s13428-011-0133-5

[21] Lynam DR, Smith GT, Whiteside SP, Cyders MA (2006) The UPPS-P: Assessing five personality pathways to impulsive behavior (Technical Report). West Lafayette: Purdue University.

[22] Cohen JD, MacWhinney B, Flatt M, Provost J (1993) PsyScope: A new graphic interactive environment for designing psychology experiments. Behavioral Research Methods, Instruments, and Computers 25: 257–271.

[23] Pinheiro JC, Bates DM (2004) Mixed-effects models in S and S-PLUS. New York: Springer.

[24] Miller J, Flory K, Lynam DR, Leukefeld C (2003) A test of the four-factor model of impulsivity-related traits. Personality and Individual Differences 34: 1403–1418.

[25] Stevens SS (1957) On the psychophysical law. Psychological Review 64: 153–181. 13441853 10.1037/h0046162

[26] Tversky A, Kahneman D (1992) Advances in prospect theory: Cumulative representations of uncertainty. Journal of Risk and Uncertainty 5: 297–323.

[27] Pleskac TJ, Wallsten TS, Wang P, Lejuez CW (2008) Development of an automatic response mode to improve the clinical utility of sequential risk-taking tasks. Experimental and Clinical Psychopharmacology 16: 555–564. 19086776 10.1037/a0014245

[28] Dougherty DM, Mathias CW, Marsh DM, Jagar AA (2005) Laboratory behavioral measures of impulsivity. Behavior Research Methods 37: 82–90. 16097347 10.3758/bf03206401

[29] Lakshminarayanan VR, Chen MK, Santos LR (2011) The evolution of decision-making under risk: Framing effects in monkey risk preferences. Journal of Experimental Social Psychology 47: 689–693.

[30] Okasha S (2007) Rational choice, risk aversion, and evolution. Journal of Philosophy 104: 217–235.

